# Introduction to the research topic: the role of physical fitness on cardiovascular responses to stress

**DOI:** 10.3389/fphys.2014.00450

**Published:** 2014-11-19

**Authors:** Daniel A. Boullosa, Arto J. Hautala, Anthony S. Leicht

**Affiliations:** ^1^Post-Graduate Program in Physical Education, Catholic University of BrasiliaÁguas Claras, Brazil; ^2^Department of Exercise and Medical Physiology, Verve ResearchOulu, Finland; ^3^College of Healthcare Sciences, James Cook UniversityTownsville, QLD, Australia

**Keywords:** physical activity, stress, exercise, cardiovascular diseases, physical fitness

This e-book is the culmination of countless hours of meticulous work by global scientists. We would like to thank the researchers for their great contributions to this hot topic. The combination of these studies reflects the importance of the topic amongst researchers and practitioners and the wide interest from numerous laboratories around the world. The contributions include a variety of formats including five original investigations, three review articles, one opinion article and a hypothesis and theory article. Notably, these contributions included both human and animal models that encompassed a range of techniques from molecular mechanisms to real life interventions thus reinforcing the translational approach for the understanding of cardiovascular responses to stress.

The three review articles (Huang et al., 2013; Carnevali and Sgoifo, 2014; Tonello et al., 2014) provided a great insight into the current knowledge of cardiovascular stress and its relationship with physical activity (PA). The first review article by Huang et al. (2013) considered the big picture of the topic by combining the classical perspective–examining how different forms of induced cardiovascular stress can be attenuated in physically trained individuals, with the addition of the interrelationships between obesity, inflammation and oxidative stress with these stress responses and cardiovascular health. The subsequent review from Carnevali and Sgoifo (2014) involved animal studies that focused on a mechanistic approach with resting vagal tone leading to stress resilience, reducing the development of anxiety and depression, and the important role of PA to mediate these relationships. The mini-review from Tonello et al. (2014) extended the topic into one of the most important social stressors in modern life, the work environment. This review revealed that factors related to adverse working conditions such as excessive effort, effort-reward imbalance, over commitment, irregular shift work, and work stress were associated with reduced cardiac autonomic function. Importantly this review identified the need for further studies in this area with heart rate variability (HRV) recognized as an important tool for evaluating both work related stress including harmful physical inactivity, and adaptations to potentially important stress buffers as PA and enhanced physical fitness.

Following the review articles, two interesting papers addressed novel and important aspects for stress management therapies. The opinion article by Stults-Kolehmainen (2013) highlighted an important and frequently forgotten aspect: How does stress negatively influence PA levels in individuals with and without cardiovascular diseases? Additionally, Stults-Kolehmainen (2013) introduced the concept of “mindfulness” for reducing stress and subsequently enhancing patients PA levels. Subsequently, Demarzo et al. (2014) discussed potentially effective means to mediate the role of physical fitness on cardiovascular responses to stress using “mindfulness” interventions that may be applied to this new area of future research.

As mentioned previously, the variety of original research investigations included within this topic reinforced the importance of research translation. The cross-sectional study by Childs and de Wit (2014) reported a significant relationship between positive affect decline after a social stressor and regular exercise in healthy individuals despite no difference between exercisers and non-exercisers for post-stress heart rate, blood pressure and cortisol responses. This study effectively illustrated how regular PA enhances psychological health and strengthens emotional resilience to stressors. Likewise, the study of Hanson et al. (2013) provided additional support for the effectiveness of regular PA in attenuating physiological responses to social stressors. Interestingly, an antidepressant drug attenuated cardiovascular responses (i.e., HR and HRV) to stress only in irregular exercisers that exhibited a comparable stress-induced response similar to that of regular exercisers during placebo (Hanson et al., 2013). These two studies (Hanson et al., 2013; Childs and de Wit, 2014) highlighted the positive impact that regular PA has on mental health, especially for depressed patients, with further studies needed to elaborate on mechanisms, benefits, and potential new therapies.

Other important responses during different sources of stress were also included in this e-book. The study of Rauber et al. (2014) was the first to our knowledge that documented post-exercise hypotension (PEH) in children. This study (Rauber et al., 2014) reported an enhanced PEH after traditional games compared to active video game playing and watching TV, and an attenuated blood pressure response during the cold pressor test following traditional games. The greater exercise intensity and metabolic demands of traditional games were highlighted as important factors for these responses with children's playing strategies fundamental for cardiovascular health.

The study of Franklin et al. (2014) documented the important contribution of other physiological responses during stress. These authors (Franklin et al., 2014) noted that endothelial function after resistance exercise (i.e., physical stressor) was impaired for obese women compared to lean women with endothelium independent-vasodilation correlated to body weight for all women. These findings provided important guidance for resistance exercise prescription of obese women to minimize cardiovascular disease risk.

Finally, the study of Sasse et al. (2013) presented a very novel hypothesis suggesting that exercise might facilitate adaptation to repeated stress via both hypothalamic-pituitary-adrenocortical axis and cardiovascular response habituation. In this study (Sasse et al., 2013), the brains of rats that performed voluntary exercise on a wheel and those that were sedentary were analyzed following control, acute and repeated noise exposures. These authors identified that unlike sedentary rats, exercising rats regulated corticotropin-releasing factor and brain derived neurotrophic factors across several brain regions. Subsequently, the hypothesis was supported with habituation to stress using exercise resulting in multiple system responses. Future studies may elaborate on the results of Sasse et al. (2013) to understand the response of different physiological systems and their interactions to stressors and applicable translation.

This e-book has taken the initial action to integrate current research findings to stimulate further discussion and research. We would like to thank the authors for their significant contributions and the many reviewers who critiqued and improved the overall topic. Future studies will clarify the importance of physical fitness, exercise and PA to regulate cardiovascular responses during stress and such benefits for cardiovascular health.

## Conflict of interest statement

The authors declare that the research was conducted in the absence of any commercial or financial relationships that could be construed as a potential conflict of interest.

## References

[B1] Carnevali L. Sgoifo A. (2014). Vagal modulation of resting heart rate in rats: the role of stress, psychosocial factors, and physical exercise. Front. Physiol. 5:118. 10.3389/fphys.2014.00118 24715877 PMC3970013

[B2] Childs E. de Wit H. (2014). Regular exercise is associated with emotional resilience to acute stress in healthy adults. Front. Physiol. 5:161. 10.3389/fphys.2014.00161 24822048 PMC4013452

[B3] Demarzo M. M. P. Montero-Marin J. Stein P. K. Cebolla A. Provinciale J. G. García-Campayo J. (2014). Mindfulness may both moderate and mediate the effect of physical fitness on cardiovascular responses to stress: a speculative hypothesis. Front. Physiol. 5:105 10.3389/fphys.2014.00105 PMC3971190 24723891

[B4] Franklin N. C. Ali M. Goslawski M. Wang E. Phillips S. A. (2014). Reduced vasodilator function following acute resistance exercise in obese women. Front. Physiol. 5:253. 10.3389/fphys.2014.00253 25071598 PMC4083188

[B5] Hanson C. S. Outhred T. Brunoni A. R. Malhi G. S. Kemp A. H. (2013). The impact of escitalopram on vagally mediated cardiovascular function to stress and the moderating effects of vigorous physical activity: a randomized controlled treatment study in healthy participants. Front. Physiol. 4:259. 10.3389/fphys.2013.00259 24069000 PMC3781330

[B6] Huang C.-J. Webb H. E. Zourdos M. C. Acevedo E. O. (2013). Cardiovascular reactivity, stress, and physical activity. Front. Physiol. 4:314. 10.3389/fphys.2013.00314 24223557 PMC3819592

[B7] Rauber S. B. Boullosa D. A. Carvalho F. O. de Moraes J. F. V. N. de Sousa I. R. C. Simões H. G.. (2014). Traditional games resulted in post-exercise hypotension and a lower cardiovascular response to the cold pressor test in healthy children. Front. Physiol. 5:235. 10.3389/fphys.2014.00235 25009506 PMC4069719

[B8] Sasse S. K. Nyhuis T. J. Masini C. V. Day H. E. W. Campeau S. (2013). Central gene expression changes associated with enhanced neuroendocrine and autonomic response habituation to repeated noise stress after voluntary wheel running in rats. Front. Physiol. 4:341. 10.3389/fphys.2013.00341 24324441 PMC3839297

[B9] Stults-Kolehmainen M. A. (2013). The interplay between stress and physical activity in the prevention and treatment of cardiovascular disease. Front. Physiol. 4:346. 10.3389/fphys.2013.00346 24348424 PMC3841719

[B10] Tonello L. Rodrigues F. B. Souza J. W. S. Campbell C. S. G. Leicht A. S. Boullosa D. A. (2014). The role of physical activity and heart rate variability for the control of work related stress. Front. Physiol. 5:67. 10.3389/fphys.2014.00067 24600407 PMC3931195

